# Testing theory of change assumptions of health behavior change interventions: A blended approach exploring local contexts

**DOI:** 10.1016/j.evalprogplan.2023.102258

**Published:** 2023-06

**Authors:** Avishek Hazra, Jaleel Ahmad, P.S. Mohanan, Supriya, Raj Kumar Verma, Sanjeev Sridharan

**Affiliations:** a Population Council, India; b Development Practitioner, India; c University of Hawai]i at Mānoa

**Keywords:** Theory of change, Testing assumptions, Self-help group, Behavior change communication, Local context and norms, Co-learning workshops and community dialogs

## Abstract

This paper used a blended approach that involves multiple techniques to, first, test a set of assumptions around a health behavior change communication intervention theory of change (ToC) and, second, surface some unidentified assumptions involving the local context. The intervention was integrated with women’s self-help groups (SHGs) in Uttar Pradesh, India. The key assumption tested in this paper was the linkage between SHG membership, program exposure, and maternal, newborn, and child health practices. Learnings were substantiated through empirical investigations, including structural equation modeling and mediation analysis, as well as ‘co-learning’ workshops within the community. The workshops aimed to capture and interpret the heterogeneity of local contexts through deep dialogs with the community and program implementers at various levels. Statistical analyses indicated a significant association between the amount of women’s program exposure and their health practices. SHG membership was shown to affect maternal health practices; however, it did not have a direct effect on neonatal or child health practices. The ‘co-learning’ workshops revealed crucial aspects, such as prevailing socio-cultural norms, which prevented pregnant or recently delivered women from participating in SHG meetings. This paper encourages evaluators to work with the community to interpret and co-construct meaning in unpacking the contextual forces that seldom appear in the program ToC.

## Highlights

• Contextual factors and social norms play important role in program theory of change.
• Unpacking the local context is essential while testing theory of change assumptions.
• Co-learning workshops and community dialogs help surface contextual heterogeneity.
• Program ToCs are dynamic and to be revisited at different phases of implementation.
• Evaluators must integrate such approaches to assess programs and interpret findings.

## 1 Introduction

Over time, the role of the program theory of change (ToC) in developing, implementing, and evaluating an intervention has received greater emphasis (Alter and Egan, 1997, Mayne, 2015, Breuer et al., 2015). A ToC describes how change is expected to happen or how change has happened (Mayne, 2017) and guides the evaluator in making choices around when and how to measure these elements of change (Connell & Kubisch, 1998). Most program ToCs specify links between input, output, and outcomes, and can potentially help strengthen the scientific case for attributing the change in outcomes to the activities included in the intervention. However, this strategy rarely includes well thought measures of ‘local contexts’ that might matter in the explanations for a particular outcome. Realist evaluators have argued that both contexts and mechanisms are fundamental in the generation of outcomes (Pawson & Tilley, 1997). Often, the ToC is developed mechanically with an assumed set of pathways. Moreover, ToCs are frequently influenced strongly by external perspectives, including project partners and donor agencies. For these and other reasons, ToCs often fail to acknowledge the dynamic nature of the change processes that evolve during program implementation. Further, the local context, such as human resources (e.g., sufficiency and capacity), the cultural setting, social norms and beliefs, and the political landscape in which the program operates, often gets neglected. While theoretical assumptions guide the choice of intervention and systematic assessment of the effectiveness of the program (Savaya & Waysman, 2005), the assumptions built into a ToC are often injected without appropriate dialogs with the program clients whose change narratives the theory of change should attempt to model.

Using an example of a health behavior change communication intervention implemented through women’s self-help groups (SHGs) in rural India, this paper tested an assumption of the program ToC using statistical techniques and then used an approach of engaging with the community and program implementers to identify the ‘unidentified’ assumptions related to the local context.

## 2 The intervention and program context

Based on existing evidence regarding the barriers to strong maternal, newborn, and child health practices (Kumar et al., 2008, Khan et al., 2012), and considering the potential of community mobilization in improving health behaviors (Manandhar et al., 2004; Tripathy et al., 2010; Baqui et al., 2008; Azad et al., 2010), in 2012 a health behavior change communication intervention program was designed to improve maternal, neonatal, and child health practices with implementation through women’s self-help groups and their federations in rural Uttar Pradesh, India. Each group consists of 10–12 women who meet every week to discuss their micro-finance activities. The SHGs and their federations were managed by a non-governmental, rights-based organization to empower women socially and economically. The microfinance-based SHG model was built on a self-help, voluntary, three-tiered approach, and was working at scale with about 1.7 million poor women across 49 districts of the state (Hazra et al., 2020).

The program operated in a very diverse and complex socio-cultural setting. The state of Uttar Pradesh has a population of more than 200 million, consisting of 75 districts and 822 development blocks, and an agriculture-based economy (Government of Uttar Pradesh, n.d.). It has four major regions based on cultural and economic diversity, with language, dialect, and culture varying even within a single district. Uttar Pradesh has seen limited shift in gender inequality over the last few decades – women are restricted to their homes, carry most of the household burden, and also contribute to the family occupation. Social groups are segregated based on caste and religion. Most of the poor and marginalized concentrate in one portion of the village. Compared to other states in India, the health indicators of Uttar Pradesh are also on the low side.

Guided by the assumption that health behavior change communication intervention, delivered through groups and federations, would improve health behaviors, a program ToC was prepared to guide program implementation. Evaluation plans were also designed at the beginning of the project, in 2012. Based on the ToC, an implementation model was prepared to reach the target women who were pregnant, had recently given birth, or had a child less than 2 years of age – through different mediums or channels with information on correct health practices. The program had eight operational regions covering 203 administrative blocks of Uttar Pradesh. Among these, the health intervention was implemented in 120 blocks and the remaining 83 blocks did not receive the health intervention. The evaluation included sampled blocks from the 120 intervention blocks with both SHGs and a health behavior change communication intervention integrated within the SHGs, as well as from the 83 comparison blocks with SHGs but without the health behavior change communication intervention.

The interventions were designed to shift community norms toward health behavior change through three sets of activities. First, discussion on maternal, newborn, and child health messages in SHG meetings by trained peer educators at least once every month. Second, interpersonal communication with target women and their family members through identification of target women and providing health information through home visits. Each SHG member was supposed to identify target women in their neighborhood and share their names in the group meetings. Eventually, each SHG member was connected with at least one target woman, acting as a friend to provide information and support on correct maternal, neonatal, and child health practices. This member was called ‘Attach-a-Sakhi’, who was supposed to do home visits to provide health information at the family level. The third set of activities included community meetings and outreach activities such as Godhbharai (a ceremony to celebrate pregnancy) and distribution of letters and leaflets containing health messages to target women. These activities were actively facilitated and arranged by the federations of the groups at the village level using audio-visual aids, such as health video shows (Hazra et al., 2020). Women’s participation in any of the above activities is referred to, in this paper, as ‘program exposure’.

In the comparison blocks, groups were engaged in micro-finance activities but discussion on maternal and child health was not part of their activities. However, the provision of normal health services from frontline health workers was common in both intervention and comparison blocks.

## 3 Key assumptions of the program ToC under testing

To illustrate the importance of the methodology in testing the ToC, we focus on two key assumptions of the program ToC – namely, that the health behavior change communication activities through the SHGs and their federations improve women’s knowledge and practices around maternal, neonatal, and child health in the SHG families. The first assumption was that the SHG members would follow and share the messages received from the discussion on health in the group meetings with their families and neighbors. The second assumption was that the home visit strategy would increase interpersonal communication with target women and their family members. Further, the program assumed that outreach activities would increase awareness in the community and create an enabling environment around correct maternal, neonatal, and child health practices.

The key assumptions of the ToC we have examined here are: a) behavior change communication program activities, implemented through SHG, increase women’s exposure to maternal, neonatal, and child health information. and b) program exposure acts as a mediating factor between SHG membership and correct health practices.

## 4 Relevance of testing these assumptions

A core assumption in the ToC was that women’s exposure to behavior change communication program activities, implemented through groups and federations, would help them follow correct maternal, neonatal, and child health practices. Yet, we need to ask whether enough consideration was given to the ‘unidentified’ socio-cultural norms that have a bearing not only on the health practices themselves, but also on participation in the health programming? What assumptions were made about the unidentified time (Sridharan et al., 2006) it may take to change these norms and/or facilitate acting against prevailing norms? Did the ToC adequately account for socio-cultural diversity, even within the program area, that may influence women’s program exposure or health practices? Further, the ToC assumed that women in the SHG families would directly benefit from the program activities. It is important to carefully examine this ‘linear’ assumption. Additionally, there might be non-linear diffusion processes underlying changes in norms. If SHG members, who are the torchbearers of the program, fail to follow correct practices, despite ‘assumed’ direct program exposure, the assumption that these members will work as change agents for a more widespread behavioral shift is a potential flaw in the ToC. It is, therefore, important to develop an understanding of where the ToC’s assumptions may be incomplete, not incorporating adequate understanding of contextual practices. What are the implications for future efforts to test ToCs when the health behavior change communication interventions through women’s groups operate at scale?

## 5 Testing assumptions through a conventional approach

Typically, the program ToC that is framed at the beginning of a project gets evaluated at the end of the project. In this case, after two years of the program intervention, using two waves of quantitative primary surveys in the intervention and comparison blocks (Hazra et al., 2020), we tested the assumptions. In wave 1 of the survey, in 2015, we used data from 4615 eligible women who were currently married, 15–49 years of age, and had given birth in the 12 months prior to the survey from SHG households (i.e., households in which at least one woman was an SHG member). Thus, a respondent could be an SHG member or a non-member from an SHG household. We also used wave 1 data from 4853 SHG leaders. In wave 2, in 2017, we used data from 4250 eligible women and 3202 SHG leaders.

The key outcome variables for testing the assumptions were women’s maternal, neonatal, and child health practices as assessed in the two waves of the survey. In each of the two survey waves, for each respondent, two composite indices – one for maternal health and another for newborn/child health practices were computed. The health program exposure was an intermediate variable in the assumed linkage between SHG membership and maternal, neonatal, and child health practices. For each woman, a summative index on health program exposure was constructed using seven dichotomized variables on the program activities a woman was exposed to during her pregnancy and after childbirth. These include (1) women’s participation in SHG meetings with health discussions; (2) home visitation by SHG cadres to provide health information/advice; (3) receipt of letters/leaflets containing health messages; participation in community outreach activities, specifically attendance at (4) *Godhbharai* event, (5) *community* meeting, (6) night meeting; (7) viewing of health video shows. Women’s membership in the SHG was the key independent variable.

We used structural equation modeling (Maruyama, 1998, Ullman and Bentler, 2012) to test the ToC pathways. To examine the causal relationship between the outcome, intermediate, and independent variables, we used SHG as the unit of analysis and treated ‘change’ as the dependent variable in the analysis. For each set of the outcome (maternal, neonatal, and child health practices), intermediate (health program exposure) and independent (SHG membership) variables, we computed the mean values of each index for each SHG at each wave and then computed the ‘change’. Mediation analysis was carried out to assess the effect of the mediating variable in explaining the relationship between the independent and dependent variables (Baron and Kenny, 1986, MacKinnon et al., 2012).

By way of some contextual background on the women respondents, average age was 26 years, and the women had three children on average. Approximately, 60% of women had attended school for formal education, in 2015; this improved by 10% points, in 2017. Women’s exposure to mass media – radio, television, or newspaper – was less than 50%. The proportion of Scheduled Caste/Tribe respondents was approximately 50%. Women in the intervention area were from households with a relatively longer association with the SHG than the comparison area, both at wave 1 (36 months versus 16 months) and wave 2 (73 months versus 46 months).

In terms of women’s program exposure, approximately 60% of women in the wave 1 survey were exposed to any program activities and received health information; this increased to 64% in two years. Women’s participation in SHG meetings with health discussions reduced from 47% in 2015, to 28% in 2017. Home visits by SHG members/cadres increased from 38% in 2015, to 55% in 2017. Approximately, one-quarter of women participated in community outreach activities in 2017. The maternal, neonatal, and child health practices of women in both areas improved over time, yet the magnitude of improvement was greater in the intervention area than the comparison area for eight of the ten indicators. Analysis of these results by regions, among the blocks where the health intervention was implemented, revealed substantial variation. For example, women’s exposure to program activities and the magnitude of improvement in the maternal, neonatal, and child health practices were much higher in some regions than others. The dialog with implementers revealed that the variation in the program exposure across regions could be due to the presence of SHGs for a longer duration, the high volume of SHGs in those regions, and proximity to the program headquarter.

Findings from the structural equation modeling suggested that SHG membership had a direct and positive association with health program exposure. Further, women’s exposure to program activities had a significant positive influence on maternal, neonatal, and child health practices. Delving more deeply, we assessed the effect of program activities on each of the maternal, neonatal, and child health practices and found that various program activities affected the practices differently. For example, delivery practices and current use of any contraceptive method were not influenced by women’s exposure to any of the program activities. On the contrary, health meetings significantly and positively influenced women having at least four antenatal care visits and postnatal care within the week after delivery. Health discussions in SHG meetings seem to influence women practicing kangaroo mother care, cord care, exclusive breastfeeding, and age-appropriate child immunization. Home visits were found to be the most effective medium toward improving all the maternal, neonatal, and child health practices.

The mediation analysis confirmed the positive role of SHG membership on maternal, neonatal, and child health practices. Health program exposure emerged as the mediator explaining the association between SHG membership and maternal, neonatal, and child health practices. Approximately 34% of the overall effect of SHG membership on maternal health practices is mediated through women’s program exposure (Table 1). For neonatal and child health practices, program exposure mediates 79% of the total effect of SHG membership on neonatal and child health practices; SHG membership did not have significant independent influence on these practices.

**Table 1:** Influence of SHG membership and program exposure on maternal, neonatal, and child health outcomes.

	Maternal health (outcome)	Neonatal and child health (outcome)
SHG membership (predictor)	β = 0.141, *p* = 0.039	β = 0.041, *p* = 0.650
Health program exposure (mediator)	β = 0.154, *p <* 0.001 34% mediated through health program exposure	β = 0.342, *p <* 0.001 79% mediated through health program exposure

## 6 Exploring the local context through co-construction dialogs: implications for the ToC

Despite the positive association between women’s program exposure and maternal, neonatal, and child health practices that emerged from the statistical analyses, women’s participation in many of the activities were low. If participation were higher, an even stronger effect on health practices might have been seen. To learn more about the underlying contextual factors, we wanted to explore why, despite so much effort, did program exposure not move ‘much’? In order to shed light on such questions, we decided to engage with the community women and cadres, and staff at all levels of the program implementation team.

In February 2019, we organized a community dissemination meeting with the ground-level program staff and cadres to share the key evaluation results and the geographic variation across the eight program regions, with a particular interest in learning more about the local context and challenges faced during program implementation. We aimed to make the event informal in the hopes of creating an engaging and open space for the participants. To this end, we presented the evaluation results in the local language, Hindi, and sat together on the floor with all participants. After a gap of four months, we organized a 4-day ‘co-construction and learning workshop’ with program cadres and program managers to learn from their reflections on the evaluation results. We deliberately tried to make the interactions less formal and used several qualitative techniques, such as key informant interviews with top level program managers, focus group discussions separately with a similar set of program personnel, and engaging sessions with group activities using flipcharts, color coded postcards, sticky notes, etc. We also made field visits to interact with women and SHG leaders to learn about the positives and negatives of the program activities and program coverage from their perspectives.

Our interactions with the program community made us realize that the ToC had failed to acknowledge that a large proportion of SHG members are older women, and most of them have achieved their desired family size. The discussions revealed that despite the SHGs’ efforts to conduct meetings and invite target women to those meetings for health discussions, the prevailing socio-cultural norms acted as barriers. For example, newly married and/or pregnant women face mobility restrictions in many rural parts of the program area. The ground-level staff reported that women were not allowed to move outside of the home during pregnancy, as it is believed that some ‘evil spirit’ may harm the mother and to-be-born child. Cultural practices, such as postnatal confinement of the mother and newborn for at least six days following the birth, did not allow interaction of program functionaries with recently delivered women.

We also learned about the ‘credibility’ of the information provider. The discussion revealed that for health information and advice, families trust frontline health workers more than SHG members or Attach-a-sakhi, who they rely on more to accompany them to the health centers to access health services. In addition, the absence of other (particularly male) members in the family during home visits to target women by a SHG member defeated the purpose of the home visit in terms of sensitizing the entire family. Following this evaluation, a new ‘positive family meeting’ strategy was adopted with the specific intention of engaging family members in the health discussion during a home visit. In the same phase of the evaluation, we learned that while, in keeping with the ToC, letters and leaflets with health messages were given to the target women, in most cases those *‘parcha’* (papers) would be kept in a ‘safe’ place -- meaning they would likely never be seen or read. The community events, such as health video shows, also suffered from several logistical challenges not visualized at the beginning of the program. As the program rolled out beyond 2017, both these approaches – distribution of health leaflets and health video shows – were discarded from the program plan.

Our discussions around the geographical diversity of output and outcome results with the program managers of all the eight regions that exhibited heterogeneity of local contexts revealed many explanations. For example, the duration of SHGs were not uniform across the program areas. Recall from the summary of the quantitative results that there was substantial variation across districts in the relationship between program activities and improved maternal, neonatal, and child health practices. While some of the regions had SHGs since 2002, other regions had relatively newly formed SHGs. It seems likely that, unless the SHGs are matured and continue with their core functions, their health program activities will be hampered.

Further, it emerged from those discussions that ‘adequate’ time needs to be given to the community before they begin to accept something new. The program started with a key focus on neonatal health; later, maternal health, and, subsequently, child health was added. Newer program activities were also ‘experimented’ with by the program management. The program managers as well as community cadres mentioned that the continuous addition of health modules, newer health messages, newer program activities and approaches caused confusion among the grassroot level workers. The duration of discussions on each message was greatly reduced and reinforcement of messages was difficult.

## 7 Discussion

This paper uses a blended approach – applying techniques such as structural equation modeling and mediation analysis in testing already explicit assumptions empirically, on the one hand, while using a ‘co-learning’ approach to unpack the contextual factors explaining and more deeply understanding the ToC assumptions on the other. It illustrates that, on its own, statistical testing of the program ToC assumptions provides an incomplete picture, even if the assumed paths seem to hold true. Adopting an approach such as ‘co-learning’ that creates an open and safe space for the program functionaries at various levels is important. The community engagement process revealed certain nuances around the assumptions, which were not considered at the time the ToC was formulated yet play a critical role in explaining key paths. A ToC, which is prepared at the beginning of a program based on existing knowledge, should not be treated as not a static framework; rather, it needs to evolve organically during the implementation process. Our knowledge base should be strengthened in an ongoing way. This means going beyond statistical techniques and adopting some unconventional approaches to learn from the community as the program progresses, and using the resulting learnings to sharpen/revise program strategies or future interventions.

To understand the success or failure of a program, evaluators must engage with and listen to the voices of ground-level program staff and cadres right at the outset, at the formulation of the theory of change. And given that human behaviors and ecology are complex, insights from the community are crucial in connecting the dots in the ToC and identifying missing links in the set of assumptions. The socio-cultural context is often a neglected factor in a sophisticated program ToC. In this paper, we showed that while empirical testing of ToC supported the assumed path of SHG membership, program exposure, and maternal, neonatal, and child health practices, it failed to identify the role of socio-cultural norms that influenced women’s participation in health programming and practices. For example, norms such as restricting pregnant women to the house presumably reduced exposure to SHG-led health activities, resulting in smaller effects than expected. Defying cultural norms and traditions invites serious punitive measures, especially in a patriarchal society. The discussion on geographical variation in program results pointed to the fact that it may be unfair to assume that all the CRDCs will perform in the same way and that the magnitude of improvement will be similar. Due to the contextual heterogeneity, even a smaller amount of change in one region may represent an equivalent degree of advancement, in light of initially poorer standing to other regions. Our dialogs with community women and program managers clearly suggest that more serious measurements of socio-cultural norms and contextual diversity are needed.

The program ToC did not consider the role of time in bringing about change in social norms and creating an enabling environment for behaviors to be sustained. Organizing and mobilizing women in a hierarchical society is a challenging and daunting task. Bringing health as a priority in their life is far more challenging. The program assumed that when a large pool of women in a community start giving health information to influence the health behavior of a target population, an environment will be created to bring change in people’s attitudes and shift community norms. The SHGs and federations can act as change agents for strengthening community capital that can remove the barriers and help in the diffusion of health messages. The co-learning workshop pointed out that challenging social norms and following correct practices may work at a slower pace than assumed.

## 8 Conclusion

Undoubtedly, the ToC is a critical element in any program planning; but the ToC needs to be revisited at different phases of the program. It is imperative to measure the nuances of contextual factors that may play a crucial role in the pathways of change. Researchers and evaluators often examine and present the results of a program at an aggregate level, using sophisticated statistical techniques to test pathways. Yet, in doing so, the heterogeneities of the local context and the existing norms, and their influence on project outcomes, are frequently overlooked. If we view learning as a dynamic act, an evaluation should not stop after an initial set of statistical tests. This study shows that despite being able to test the assumed ToC paths, the robust statistical analyses failed to provide answers as to ‘why’ certain building blocks of the ToC were at different levels with variation across program geographies, or ‘how’ the assumptions followed or deviated from the ToC path. Understanding these ‘why’ and ‘how’ elements of program outputs and outcomes demands a detailed engagement with community and implementers, and a much deeper probe into local contexts.

Community dialogs and co-learning workshops facilitated a deeper engagement between the evaluators and program implementers. These approaches helped surface the heterogeneity of socio-economic and cultural context and norms in explaining the assumed paths. For large and complex programs, it is necessary to revisit the program ToC jointly with implementers, evaluators, and other partners at appropriate intervals to identify important local contextual factors that may have been missed in the initial ToC.

## 9 Lessons learned: remembering John Mayne

We are students of John Mayne, and we were influenced by the discipline he brought to thinking about theories of change – for example, his focus on different kinds of assumptions helped us formulate our research questions more clearly. Our contribution in this paper will perhaps most closely correspond to his focus on learning and building an evaluative culture (Mayne, 2009). We add to his thinking by exploring the types of methods that can help with learning from initial assumptions of theories of change.

We believe our paper highlights the following six kinds of learnings:

1) Contexts matters in learning about assumptions:
  This is not a new insight and was a focus of Mayne’s work in the last few years of his life. Approaches such as realist evaluation (Pawson et al., 2004) make context quite central to the evaluator’s work; yet, we believe that there needs to be more detailed discussion of where knowledge of context comes from. As discussed in the introduction to this volume, knowledge of context in theories of change are often incomplete, and often we need to learn about context as the implementation proceeds.
2) Lack of upfront knowledge of the timeline of change for linkages:
  A second kind of incompleteness regarding some linkages in a theory of change is the lack of knowledge of temporality. For example, most theories do not describe how long it would take an intervention to impact an outcome. Further, anticipated timelines of impact might differ across different contexts. We believe there is need for more focused discussion of temporality in the linkages of the theory of change.
3) A space for dialog with key stakeholders:
  Perhaps one of the most central lessons for us is that learning about assumptions cannot simply involve data analysis. We needed spaces for dialog, and it was important that such a space was created in a way that promoted dialog without implied asymmetries in power -- for example, we insisted that, like all participants, we also sat on the floor during the dialog. Such dialog and spaces must be created with humility and openness to uncertainty. The pretense that all knowledge resides in statistical data can interfere with interpretation and co-construction.
4) The importance of mixed methods:
  This approach also strongly reinforced for us the critical importance of mixed methods. We work in organizations that tend to prefer statistical methods over qualitative methods or methods based in dialog and co-construction. In our experience, concepts and measurement, including properties of important concepts like social norms, are often adopted on the basis of the psychometric properties. We think greater care needs to be spent in ensuring that we understand how the participants in an intervention understand concepts such as social norms. Our lesson here is that distant views of measurement should not be reified over the views of an underlying concept as the clients themselves see it. Given that the program recipients are the ones with the greatest ‘skin in the game,’ it is important to make them more central in the definition of key measures.
5) Learning needs of clients and other stakeholders:
  Another key learning that the co-construction dialog highlighted was: Who needs to learn from exploring the assumptions in a theory of change? Our experience has been that often the needs of a funder or an academic/consultancy organization are often implicitly considered higher than the needs of the program participants. In the co-construction dialogs, it became obvious that community members themselves appreciated the dialogs, and they had far deeper insights on the weaknesses of our statistical models including key gaps in our theorizing and measurement. Such knowledge only surfaced after we created spaces in which their views were treated not only as validation of the results but as a constant process of refinement and development in understanding the assumptions in a theory of change.
6) Starting with the program clients:

Finally, one of our key learnings was that it is important to conduct these dialogs up front before we complete an initial theory of change. Theories of change fundamentally inform the key constructs that define how programs work and also provide one foundation for measurement. It is important that we are informed by how key program participants view a program in both our conceptualization and measurement. Engagement with clients should not be only of refinement or validation but of creating a space in which they become the key owners of a theory of change. It is neither a funder nor an evaluation consultant who owns a theory of change, but the theory of change fundamentally reflects stories of what could happen to program recipients. Given the limited knowledge we have of participant/recipient/client contexts, it is only fair to construct such stories by first involving the participants.

## Funding

The evaluation work was supported by the 10.13039/100000865Bill & Melinda Gates Foundation, Seattle, WA [grant number: OPP1033910].

## Declaration of interests

None.
